# Oxygen Toxicity and Special Operations Forces Diving: Hidden and Dangerous

**DOI:** 10.3389/fpsyg.2017.01263

**Published:** 2017-07-25

**Authors:** Thijs T. Wingelaar, Pieter-Jan A. M. van Ooij, Rob A. van Hulst

**Affiliations:** ^1^Diving Medical Center, Royal Netherlands Navy Den Helder, Netherlands; ^2^Department of Anaesthesiology, Academic Medical Center Amsterdam, Netherlands

**Keywords:** oxygen toxicity, CNS-toxicity, pulmonary toxicity, diving, closed-circuit rebreather

## Abstract

In Special Operations Forces (SOF) closed-circuit rebreathers with 100% oxygen are commonly utilized for covert diving operations. Exposure to high partial pressures of oxygen (PO_2_) could cause damage to the central nervous system (CNS) and pulmonary system. Longer exposure time and higher PO_2_ leads to faster development of more serious pathology. Exposure to a PO_2_ above 1.4 ATA can cause CNS toxicity, leading to a wide range of neurologic complaints including convulsions. Pulmonary oxygen toxicity develops over time when exposed to a PO_2_ above 0.5 ATA and can lead to inflammation and fibrosis of lung tissue. Oxygen can also be toxic for the ocular system and may have systemic effects on the inflammatory system. Moreover, some of the effects of oxygen toxicity are irreversible. This paper describes the pathophysiology, epidemiology, signs and symptoms, risk factors and prediction models of oxygen toxicity, and their limitations on SOF diving.

## Introduction

Military diving, especially within the domain of the Special Operations Forces (SOF), is one of the most extreme forms of diving. Depending on the task, this type of diving demands different equipment, procedures and training and, therefore, it is totally unlike commercial or civilian diving. For SOF divers, range and endurance, high mobility and stealth, are of utmost importance. To facilitate these requirements, the most commonly used equipment is the closed-circuit oxygen rebreather (O2-CCR): a comprehensive overview of the historic aspects of CCR diving is already published (Donald, 1992; Acott, 1999; Butler, 2004).

A CCR is fundamentally different from any open-circuit or semi-closed diving system. Instead of releasing exhaled air to the surrounding environment, it is recirculated within the apparatus. Any exhaled carbon dioxide (CO_2_) is “scrubbed” by a chemical mixture, often various hydroxides, for instance NaOH and Ca(OH)_2_. The efficacy of scrubbers is beyond the scope of this review, but factors like granule size, ambient temperature, and humidity greatly affect and sometimes limit scrubber efficiency. CCRs can be used with air, mixed gas, or pure oxygen. There is no exhaled gas under the form of bubbles and gas consumption is very much limited, increasing the possible autonomous dive time. In case of a breathing gas composed of pure oxygen, a second substantial difference compared to a regular SCUBA or mixed gas rebreather can be noted: the oxygen diver has no accumulation of nitrogen or other inert breathing gas and, therefore, no decompression limits. To facilitate recirculation, the breathing gas is temporarily stored in a “counter lung” before inspiration. The volume of this counter lung limits the tidal volume (TV) and maximum minute volume (MMV) of the SOF diver. The limitation of TV and MMV and possible saturation of the soda lime can cause retention of CO_2_ (Arieli et al., 2006a,b). This greatly affects the development of oxygen toxicity (see below).

Although many studies have investigated the toxic effects of oxygen on both the central nervous system (CNS) and the pulmonary system, the question remains how applicable those studies are for SOF divers. Much of the research was conducted with animals; although this has greatly contributed to our understanding of physiological processes, the results cannot always be extrapolated to humans (Robinson et al., 1974; Bryan and Jenkinson, 1988; O'Collins et al., 2006). Secondly, much of the available data on humans is rather old, and many of these experiments will never be replicated today due to our current viewpoint on research ethics. Nevertheless, these historical studies give some insight into the (life-threatening) dangers that oxygen poses for man (Donald, 1992; Acott, 1999). Since technological advances (such as, the capacity of “scrubbing” CO_2_) influence the occurrence of oxygen toxicity, many of these older studies cannot be used to determine the threshold or safe limits or oxygen exposure. Lastly, most human experiments were performed in rest in a recompression chamber, the so-called “dry dives.” Although dry dives enable researchers to administer oxygen in partial pressures above 1 ATA (equal to 101.3 kPa), the effects of oxygen are not the same as in an actual dive. Several studies have shown that submersion can alter the physiologic reaction to breathing gases (Donald, 1992; Kerem et al., 1995; van Ooij et al., 2011).

However, even when taking these limitations into consideration, some relevant data on oxygen toxicity in diving are still available. The aim of this paper is to summarize the pathologic effects of oxygen (mainly on the CNS and pulmonary system) and their operational consequences for SOF divers.

## Central nervous system toxicity

The phenomenon of CNS toxicity is commonly referred to as the Paul Bert effect, named after the French physiologist who first described it (Bert, 1878). In many dry dive experiments, Bert showed that oxygen is toxic and potentially lethal for many organic species including seeds, fungi, insects, and several small mammals. Others published similar results, showing that CNS toxicity was dependent on the inspired partial pressure of oxygen (PO_2_) and the time exposed. In 1910 Bornstein was probably the first to expose two human volunteers in a dry dive setting to hyperbaric oxygen at a PO_2_ of 2.8 ATA (equal to 283.7 kPa) for 30 min without any complaints (Acott, 1999).

The tolerance for oxygen in dry dives is much higher than in wet dives (Donald, 1992). In an immersed setting, a PO_2_ above 1.4 ATA can lead to nausea, numbness, dizziness, twitching, hearing and visual disturbances, unconsciousness and convulsions (Harabin et al., 1995). In humans, no oxygen induced convulsions have been described with a PO_2_ lower than 1.3, although susceptibility to oxygen toxicity has a high interpersonal and intra-individual variability (Donald, 1992; Arieli et al., 2008). While convulsions can occur without any prior symptoms, visual disturbances generally precede convulsions (Curley and Butler, 1987; Arieli et al., 2006a). Reports on the incidence of CNS toxicity vary greatly, ranging from 1 in 157,930 CCR dives to approximately 3.5% of the CCR dives (Harabin et al., 1995; Walters et al., 2000; Arieli et al., 2002). This may be attributed to different exposures in time and depth, or to different definitions of the symptoms or, perhaps, because the covert nature of SOF diving precludes the reporting of precise incidences.

### Pathogenesis and risk factors

Although the exact mechanism is not fully understood, currently, the most plausible explanation is related to an overflow of reactive oxygen species (ROS) in the brain after an increase of cerebral blood flow (CBF) (Visser et al., 1996a; Koch et al., 2008). Due to the increased PO_2_ in plasma there is an auto oxidation of nitric oxide (^·^NO) to several ROS, of which peroxynitrite (ONOO^−^) is the most important (Goldstein and Czapski, 1995; de Groot et al., 2004). ROS cause angiotensin-II induced vasoconstriction via activation of non-phagocytic NAD(P)H oxidase (Weissmann et al., 2000; de Groot et al., 2004; Nguyen Dinh Cat et al., 2013). However, at the same time, endothelial and neuronal nitric oxide synthase (eNOS and nNOS), which are responsible for vasodilatation, are increased (Hoehn et al., 2003). The net result of both processes is vasoconstriction and a reduction of CBF up to a certain “breaking point.” Possibly due to depletion of the radical oxygen scavenger system, vessels dilate and increases CBF (Chavko et al., 1998; Demchenko et al., 2002; Eynan et al., 2014). Simultaneous to this increase in CBF, the cortical electroencephalography (EEG) activity increases (Bean and Coulson, 1971; Visser et al., 1996b). This increase in CBF and cortical EEG activity precedes convulsions (Bean and Coulson, 1971; Visser et al., 1996a,b; Demchenko et al., 2001; Koch et al., 2008). The exact mechanism though which ROS cause convulsions is not entirely clear. ROS are believed to directly affect various ionic conductance that regulate cell excitability, as well as disrupting chemical synaptic transmission (Manning, 2016). The role of superoxide dismutase (SOD), catalase and other scavengers in the brain, predominantly on the function of the hippocampus, remains to be elucidated. However, animal experiments have shown that modulating the N-methyl-D-aspartate (NMDA) and N-nitro-L-arginine (NNA) system alters the susceptibility (Eynan et al., 2014; Manning, 2016). When any prodromal symptoms are encountered and the PO_2_ is lowered, convulsions may be avoided (Arieli et al., 2008).

Despite extensive research to identify risk factors in CNS toxicity, most studies were on animals and the relevance for oxygen diving in humans remains unclear. Oxygen toxicity is dependent on both PO_2_ and time, i.e., the time of onset of symptoms is shorter when the PO_2_ is higher (Donald, 1992; Arieli et al., 2002). An increase in end-tidal partial pressure of carbon dioxide (PetCO_2_), either by “CO_2_ retainers” (divers with a delayed or altered response to hypercapnia) or due to exercise, also increases susceptibility to oxygen toxicity (Arieli et al., 2001; Koch et al., 2013). Dehydration and starvation prolongs the latent period to onset of convulsion in rats, but the pathophysiological mechanism is unclear (Bitterman et al., 1997). In a small study, a ketogenic diet in divers increased oxygen tolerance, but the mechanism for neuroprotection remains unknown (Valadao et al., 2014). Adding nitrogen or helium to the inspired gas, as well as adding periods of breathing air (commonly called “air breaks”) to the oxygen exposure, protect against convulsions in dry dives, but the feasibility in oxygen diving is limited (Hendricks et al., 1977; Bitterman et al., 1987; Harabin et al., 1988; Arieli et al., 2005, 2008). An overview of pharmacological agents and vitamins that protect or sensitize has recently been published (Jain, 2017). A few pharmacological agents are worth mentioning here: scopolamine and cinnarizine (agents frequently used to prevent and treat motion sickness) do not seem to either attenuate or sensitize oxygen toxicity (Bitterman et al., 1991; Arieli et al., 1999). Caffeine is effective in delaying convulsions in rats, but its efficacy in humans has yet to be confirmed (Bitterman and Schaal, 1995). Due to the unpredictability and operational limitations, relying on pharmacological agents for extending SOF diving is not currently relevant.

### Prediction model and variability

The first prediction model (published by Harabin et al.) was based on 661 CCR dives (Harabin et al., 1995). This was later refined by Arieli et al. who based their model on 2,039 CCR dives and which remains the most accurate model to date (Arieli et al., 2002). The chance of oxygen toxicity (as Z-score in a normal distribution) in any dive can be estimated by: *Z* = [ln(*t*) −9.63 + 3.38 × ln(PO_2_)]/2.02. Note that this equation includes PO_2_ (in kPa) and time (in min) as variables. The recovery time (similar to “surface interval” in air dives: the time for a diver to neutralize the oxygen stress) is based on experiments in rats and estimated by: K_*t*_ = K_e_ × *e*^−0, 079*t*^, where K_*t*_ is regarded as a cumulative oxygen toxicity index at time *t* (in min) and K_e_ the “toxicity dose” at the end of exposure. However, to our knowledge, no studies have tested the efficacy of these models in humans.

Although the methodology of the model is sound, a considerable intra and interpersonal variability in oxygen toxicity still remains. In an effort to identify the military divers at risk, the “oxygen tolerance test” has long been advocated, where subjects were exposed to breathing 100% oxygen at 2.8 ATA for 30 min (Butler and Knafelc, 1986). However, after evaluation, this was proven obsolete because it lacked predictive value and many navies now refrain from using this test (Visser et al., 1996b; Walters et al., 2000). A similar test for CO_2_, the Read test, has also proven ineffective (Arieli et al., 2014). Although the ability to detect CO_2_ by divers can be trained, it is unknown whether this reduces the incidence of oxygen toxicity (Eynan et al., 2003, 2005). To our knowledge no valid test is available to screen for oxygen tolerance.

### Operational consequences

Even though the pathophysiological mechanisms and risk factors are not yet clarified, there is a clear depth (PO_2_) and time relationship. Oxygen toxicity of the CNS is a rare but potentially life-threatening complication of exposure to high PO_2_, which can occur without prodromal symptoms. If mild symptoms do occur and can be timely recognized, convulsions may be avoided by reducing depth. However, delayed progression to convulsions after reducing oxygen exposure have been described.

In sports diving, the PO_2_ limit recommendations ranges from 1.4 to 1.6 ATA (Lang, 2001). The limits for military SOF diving are different, due to differences in the equipment and the amount of “acceptable risk” (Table 1) (Vann, 1988; United States Department of the Navy NSSC, 2008). The model from Arieli et al. allows 24 min at a PO_2_ of 2.5 ATA when accepting a risk of maximum 5% of CNS toxicity (Arieli et al., 2002). The widely used US Navy Diving Manual allows a single-depth exposure to a PO_2_ of 2.5 ATA for 10 min (United States Department of the Navy NSSC, 2008). However, these high risks are only taken with the proper training, equipment and safety precautions, and only when the operational demands leave no alternative options. Incidence reports of oxygen toxicity using the Arieli model are lacking, most likely due to the covert nature of SOF diving.

**Table 1:** Single-depth oxygen exposures.

**Depth (fsw/msw)**	**Limit US Navy (United States Department of the Navy NSSC, 2008) (min)**	**Risk of CNS toxicity (Arieli et al., 2002)(%)**	**Limit Arieli et al. <5% (min)**
25/7.7	240	13.1	83
30/9.2	80	6.3	63
35/10.7	25	2.4	48
40/12.3	15	1.7	38
50/15.3	10	1.8	24

*Depth in feet of sea water (fsw) and meters of sea water (msw). The limits are printed as in the US Navy Diving Manual with (in the third column) their associated risk for CNS toxicity based on the model of Arieli et al. (2002). The last column shows the bottom time when accepting a maximum risk of CNS toxicity of 5%*.

## Pulmonary oxygen toxicity

In 1899, the Scottish pathologist, James Lorrain Smith, published the pathological effects of increased inspiratory oxygen tension on several small animals (Lorrain Smith, 1899). In these classic experiments, mice and larks were exposed to increasing pressures of oxygen for long periods of time. Besides several episodes of CNS toxicity, most of the animals perished because of hypoxia as a result of insufficient ventilation due to acute or chronic lung inflammation. Compared to CNS toxicity, a lower partial pressure of oxygen is required to cause symptoms, but the exposure time has to be much longer (hours to days). Exposure above 0.5 ATA is regarded as potentially damaging for the pulmonary system. In humans, early symptoms include tracheobronchial irritation with retrosternal pain and coughing (Klein, 1990). Longer exposures damage the tracheal mucosa with impaired mucus clearance (Sackner et al., 1975). These complaints precede changes in lung function tests, such as, a decrease in vital capacity (VC), but have a low predictive value due to high variability (Klein, 1990). The incidence in divers is unknown, since no studies have investigated the epidemiology.

### Pathophysiology and risk factors

**Figure 1:**
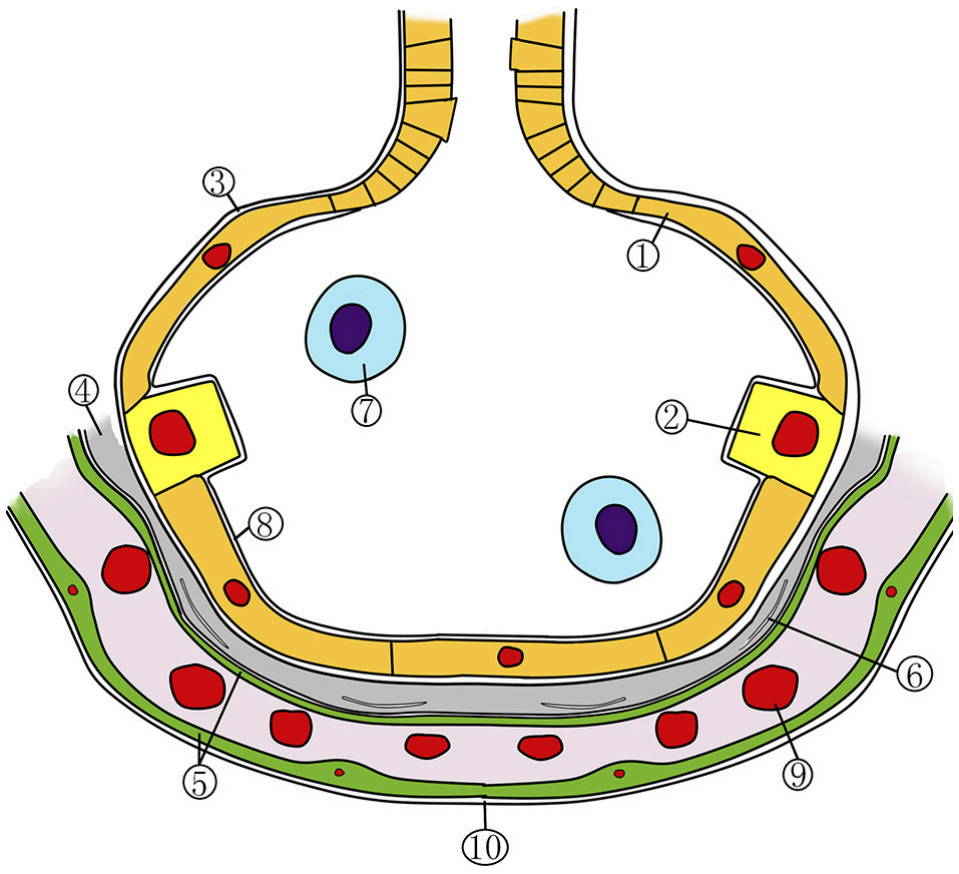
Schematic representation of the normal alveolocapillary region. 1, alveolar type 1 cell; 2, alveolar type 2 cell; 3, basement membrane; 4, interstitium; 5, capillary endothelial cell; 6, fibroblast; 7, alveolar macrophage; 8, surfactant layer; 9, red blood cell; 10, capillary base membrane. Adapted with permission from van Ooij et al. (2013).

**Figure 2:**
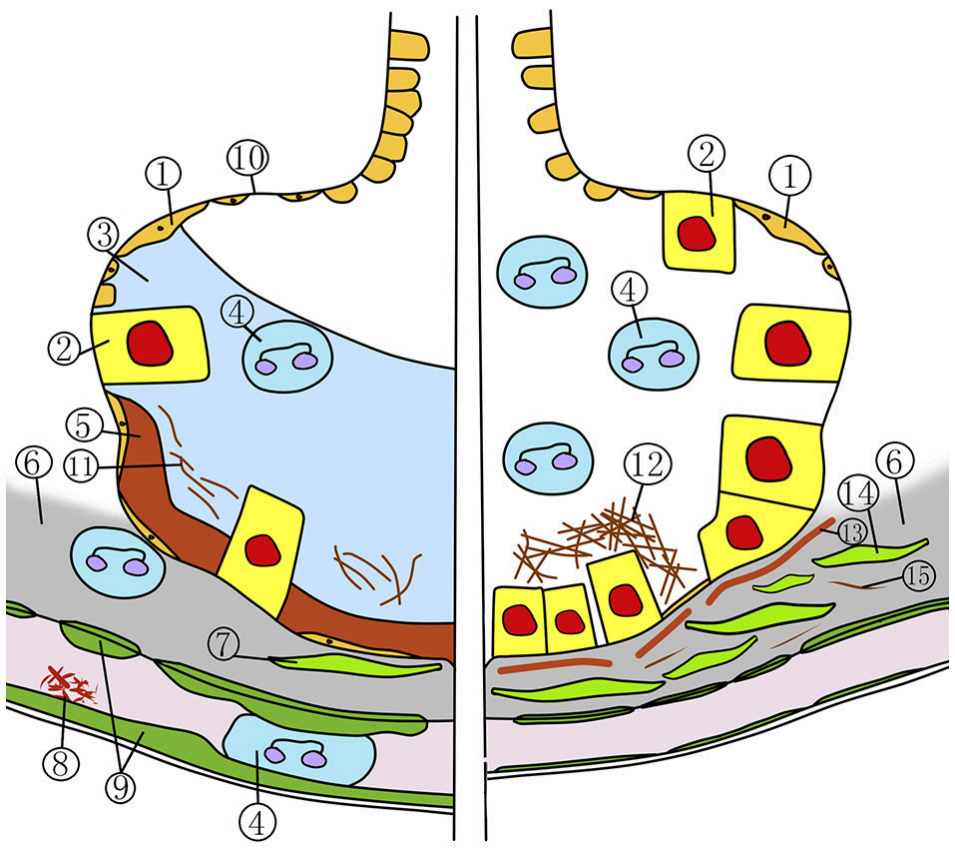
Exsudative stage (left) and proliferative stage (right) in pulmonary oxygen toxicity. 1, type 1 alveolar cell; 2, type 2 alveolar cell; 3, alveolar edema; 4, neutrophil; 5, hyaline membrane; 6, edematous interstitium; 7, fibroblast; 8, fibrin thrombus; 9, swollen capillary endothelial cell; 10, denuded basement membrane; 11, alveolar fibrin formation; 12, collagen fibers deposition; 13, incorporation of hyaline membrane; 14, fibroblastic proliferation; 15, interstitial fibrin.

Pulmonary oxygen toxicity (POT) can be divided into two phases. The first exsudative phase (**Figure 2**, left side) is marked by local inflammation with capillary and endothelial edema, a decrease of type I alveolar cells, and an influx of inflammatory cells (Miller and Winter, 1981; Bryan and Jenkinson, 1988; Demchenko et al., 2007). These changes are reversible and the lung returns to its normal state (Figure 1) if the inspired oxygen pressure is reduced below 0.5 ATA. In the following proliferative phase (Figure 2, right side) fibroblasts and type II alveolar cells infiltrate the inflamed endothelia. With continuing inflammation, this ultimately leads to alveolar fibrosis and a four- to fivefold increase of thickness of the air-blood membrane and, as a consequence, loss of diffusion capacity (Kapanci et al., 1972; Robinson et al., 1974). These changes are irreversible. The rate at which these changes occur is directly related to the inspired PO_2_ and can occur as early as 3 h at a PO_2_ of 3 ATA during a dry dive (Winter and Smith, 1972; Klein, 1990).

When divers are immersed, many physiologic processes are altered. Circulating volume is redistributed due to the hydrostatic pressure on the body and peripheral vasoconstriction when immersed in cold water, both resulting in volume shift and intrathoracic pooling (Norsk et al., 1985; Choukroun et al., 1989; Pendergast and Lundgren, 2009). Even though the mammalian diving reflex lowers the heart rate, the net result of both processes is pulmonary hypertension, because the cardiac output is increased as a result of the Frank-Starling mechanism (Dahlback et al., 1978). The increased blood flow in the lung recruits apical fields (compared to exercise), but also stiffens the lung (Choukroun et al., 1983; Pendergast and Lundgren, 2009). Beside these effects on lung circulation, the intrathoracic pooling and pulmonary hypertension triggers the baroreceptors in the right atrium, which increases diuresis through an increased vasopressin release (Norsk et al., 1986; Boussuges et al., 2007, 2009). Lastly, the position of the diver in the water (horizontal or vertical) and the position of the breathing apparatus compared to the body (deeper or lower than the diver) also influences perfusion of the lung and breathing dynamics (Badeer, 1982; Taylor and Morrison, 1991). As a result of all of the above processes, gas exchange in the lung during immersion is substantially different from that during dry dives (Prefaut et al., 1978; Taylor and Morrison, 1990; van Ooij et al., 2011).

Very few studies have reported risk factors for developing POT in divers, let alone in SOF divers. Most of the results are derived from dry dive experiments. Many of these studies use a decrease in VC as a marker to determine the amount of POT; however, the validity of this measurement is questioned (see below). Shykoff reported that exercise in an immersed setting and repeated exposure increases POT (Shykoff, 2008a,b). To our knowledge, no other risk factors have been identified. In animal studies, scavengers (such as, SOD and catalase) were shown to protect lung tissue against the overload of oxygen radicals; however, this effect has not been confirmed in humans (Kimball et al., 1976; Frank et al., 1978; Potter et al., 1999).

### Prediction model and variability

In military and commercial diving, the current standard for determining the maximum pulmonary oxygen exposure in diving is “units of pulmonary toxicity dose” (UPTD). One UTPD equals the amount of damage caused by breathing 1 min of 100% oxygen at 1 ATA (Bardin and Lambertsen, 1970). The basic concept of UPTD is that a certain threshold (amount of oxygen molecules) is required to cause local damage, which can be measured by a decrease in VC. For instance, an exposure of 615 UPTD causes the VC to decrease 2% in 50% of the divers, while 1425 UPTD lowers the VC by 10% in 50% of the divers (Clark and Lambertsen, 1970; Wright, 1972). Since the 1970s, many studies have further refined the basic model (Clark et al., 1999). To calculate the amount of UPTD, the following equation must be solved: UPTD = *t* × [0.5/(PO_2_−0.5)]^−5/6^, with PO_2_ in ATA and time in minutes. Arieli et al. published an improved equation to more accurately determine the decrease in VC in a dry setting, based on data from several studies that included exposures with humans: ΔVC = 0.0082 × *t*^2^(PO_2_/101.3)^4.57^; please note that, here, PO_2_ is in kPa and time is in hours (Eckenhoff et al., 1987; Clark et al., 1991; Arieli et al., 2002). The “cumulative units of pulmonary toxicity dose” (CPTD), the “oxygen toxicity unit” (OTU) and derived equation for repetitive exposure (REPEX) were introduced to include recovery and facilitate multi-day exposures, but was never validated in divers (Hamilton, 1989). Arieli et al. also published an updated equation to estimate the recovery of lung volume, which was extrapolated from data derived from animal experiments performed in a dry setting (Arieli et al., 2002). There is no consensus which model is the most valid to plan SOF operations.

The main flaw in the UPTD concept and the derived equations is the change in VC as the sole indicator to determine oxygen stress. VC has a circadian rhythm and there is a strong intra and interpersonal variability when measuring lung volumes (Hruby and Butler, 1975; Harabin et al., 1987). Ventilation during anaesthesiology with a high PO_2_ is known to influence VC, possibly due to absorption atelectasis (O'Brien, 2013). Whether this also occurs in SOF divers, or how long this endures after diving, is unknown. Recent findings have proven that immersion itself alters VC regardless of oxygen stress (Shykoff, 2005; van Ooij et al., 2011, 2012). Since the UPTD model was derived from dry dives, the above-mentioned factors are not taken into account. Although the original authors recognized the limitations of the UPTD model, more advanced diagnostic measurements were either too difficult to perform or were unavailable in the 1960s/1970s (Bardin and Lambertsen, 1970).

### Operational consequences

POT is more insidious than CNS toxicity; it affects the oxygen divers in long shallow-water dives or when recurrently exposed. The current prediction model (UPTD) was developed in dry setting during a time when capabilities to measure lung parameters were limited. Newer parameters, such as, the ratio between diffusion capacity of carbon monoxide and nitric oxide (DL_NO/CO_), fraction of exhaled nitric oxide (FE_NO_) or volatile organic compounds (VOCs), might be more accurate in determining POT, but these tests have yet to be validated (Shykoff, 2008a,b; Caspersen et al., 2011; van Ooij et al., 2014b,a, 2016; Vermeulen et al., 2016). Especially the VOCs are of interest because, in the field of pulmonology, this noninvasive diagnostic modality is increasingly utilized for diagnosing asthma, acute respiratory distress syndrome and lung cancer (Bos et al., 2014, 2016; Boots et al., 2015). However, until a new valid parameter to determine POT has been established, the UPTD model remains the gold standard, despite its limitations.

As equipment improves and dive times are extended, SOF divers might be increasingly exposed to a level at which irreversible damage may occur. The Royal Netherlands Navy currently dives with the LAR 5010 by Dräger and within the limits given by NATO (Allied Diving Publication), which are highly similar to the US Navy Diving Manual (United States Department of the Navy NSSC, 2008). Oxygen exposure is limited to 450 UPTD per day and 2250 UPTD per week. A single exposure up to 1425 UPTD is regarded the absolute maximum and only to be used in exceptional circumstances with sufficient medical support available (i.e., recompression facilities and medical capacity within the operational theater). The Royal Netherlands Navy Diving Medical Center performs yearly dive medicals on all Dutch SOF divers according to and surpassing the standard of the European Diving Technology Committee (Wendling and Nome, 2004). In a recent 20-year longitudinal cohort study, we found no significant changes in pulmonary function and diffusion capacity of SOF divers compared to other Navy divers or non-divers (Voortman et al., 2016). Tetzlaff et al. published similar results (Tetzlaff et al., 2005). Yearly exercise tolerance testing shows VO_2 max_ values regularly surpassing 50 ml/kg/min and all divers remain fit for diving duties during their career. This may be due to either sufficient “recovery” time between extreme dives, or because exposures are not severe enough to cause irreversible damage. Although current monitoring does not show any deleterious effects, it remains necessary to continue this monitoring of long term health effects as the level of exposure in recent years has increased.

## Other pathophysiologic changes

While CNS toxicity and pulmonary toxicity have been described as separate entities in this review, their occurrence may be more closely related. In addition to cold, stress and physical activity, CNS toxicity activates the sympathetic nervous system, which in animal experiments leads to pulmonary edema though the pulmonary venule adrenergic hypersensitivity response (Winklewski et al., 2013). Hyperoxia, even at normobaric conditions, induces many physiological changes which are often not fully understood. In addition, the clinical relevance of these changes and impact on SOF diving remains to be elucidated. Although this paper does not aim to give a full review of all known pathophysiological effects of oxygen in divers, the effects on sight and exercise tolerance are important in the context of SOF diving. For further reading of the effects of hyperoxia on other parts of the body we suggest the work of Bennett and Elliott (Brubakk and Neuman, 2003).

### Ocular toxicity

Visual acuity is of crucial importance to SOF divers. Visual complaints are a frequent side-effect of daily clinical treatments in recompression chambers (hyperbaric oxygen therapy: HBOT). Transient myopia with up to 0.25 dioptres loss for each week exposed to high oxygen pressures can occur, but generally resolves after a few weeks (Butler et al., 2008). Apart from one case report, hyperopic myopia has not been reported in oxygen divers (Butler et al., 1999). Although extreme HBOT exposures can cause irreversible cataract or keratoconus, this has not been described in divers (Palmquist et al., 1984; Butler, 1995; McMonnies, 2015). These effects of oxygen on the ocular system are probably irrelevant for SOF divers, as oxygen pressures are generally much lower and exposure is less frequent compared with daily HBOT in patients for several weeks.

### Exercise tolerance

There are several reports on fatigue and reduced exercise tolerance after high oxygen exposures (Comroe et al., 1945; Lambertsen, 1978; Shykoff, 2005). Divers complained about retrosternal pain or the inability to “give their full” for several days. To what extent this is a subjective complaint, or limits (diving) performance, is unknown. Although the mechanism behind these complaints is not fully understood, generalized oxidative stress depletes the scavenger system and leads to lipid peroxidation of the cell membranes causing cell damage (Ferrer et al., 2007; Perovic et al., 2014). After diving, because there is an upregulation of glutathione peroxidase (GPx) and catalase activity in lymphocytes, the inflammatory system may also be involved (Ferrer et al., 2007). Damage and dysfunction of erythrocytes has been described after hyperbaric hyperoxic exposure and in saturation divers, its effect on exercise tolerance is unknown (Dise et al., 1987; Hofsø et al., 2005). To what extend performance is impaired in SOF divers after oxygen diving remains to be confirmed.

## Summary

In diving and hyperbaric environments, oxygen toxicity has been a topic of interest for over a century. Although many human experiments are not reflecting current equipment or procedures anymore, the results do illustrate the damaging potential of oxygen. Diving with high partial pressures of oxygen can result in acute life-threatening neurologic complications or irreversible pulmonary structural changes. However, the extent to which these problems occur in oxygen diving remains unknown, due to the lack of studies on humans during immersion, and/or epidemiologic studies.

In SOF diving, where 100% oxygen rebreathing diving systems are frequently used, operational demands and health risks are taken into account when planning dives. All current limits or diving tables with high PO_2_ possess a certain quantity of “acceptable” risk. The question arises as to whether civilian or commercial divers should use the same limits as SOF divers.

To develop more accurate prediction models, we need to identify the pathophysiological mechanism of oxygen toxicity and the factors that, subsequently, increase or decrease the risk to various parts of the body. This is complicated by the covert nature of SOF diving, limiting publication of data. Also, in view of the considerable inter- and intra-personal variability, perhaps the future of oxygen diving requires real-time individual monitoring of early symptoms of oxygen toxicity, such as, CBF or exhaled VOCs, to protect humans from the harmful effects of oxygen when diving.

### Current limits on oxygen exposure in the royal netherlands navy

#### Central nervous system oxygen toxicity

Divers exposed to a PO_2_ above 1.3 ATA should be considered to be at risk for developing CNS toxicity. An estimation of the chance of CNS toxicity in diving, as *Z*-value in a normal distribution with *t* in minutes and PO_2_ in kPa, can be made (Arieli et al., 2002). There is no consensus regarding a “maximum acceptable risk.”

(1) $$Z=\frac{\text{ln}(t)-9.63+3.38\times \text{ln}({\text{PO}}_{2})}{2.02}$$

#### Pulmonary oxygen toxicity

Any PO_2_ above 0.5 ATA is regarded as toxic for the pulmonary system. The amount of “units of pulmonary toxicity dose” (UPTD), with *t* in minutes and PO_2_ in ATA, can be calculated with the function below (Bardin and Lambertsen, 1970). Many authorities regard an exposure of 615 UPTD as the “maximum safe exposure for a single dive”.

(2) $$UPTD=t^{-1.2}\text{​}\sqrt{\frac{0.5}{{\text{PO}}_{2}-0.5}}$$

## Author contributions

TW student of RvH: Acquisition and review of literature, drafting, and revising manuscript. PJvO co-promotor of TW: Review of literature, help with theoretical framework, writing, and reviewing concept manuscripts. RvH promotor of TW: Review of literature, help with theoretical framework, writing, and reviewing concept manuscripts.

### Conflict of interest statement

The authors declare that the research was conducted in the absence of any commercial or financial relationships that could be construed as a potential conflict of interest.
